# Comment prendre le pas sur le coronavirus dans un pays en développement: questions et actions au Burkina Faso

**DOI:** 10.11604/pamj.supp.2020.35.2.23033

**Published:** 2020-05-15

**Authors:** Emmanuelle Semporé, Herman Bazié, Bernard Ilboudo, Hervé Kpoda, Blandine Bila, Télesphore Somé, Olivier Sossa, Clément Méda, Hervé Hien

**Affiliations:** 1 Institut National de Santé Publique (INSP), Ouagadougou Burkina Faso; 2 Institut de Recherche en Sciences de la Santé (IRSS), Ouagadougou, Burkina Faso; 3 Société d’Etude et de Recherche en Santé Publique (SERSAP), Ouagadougou, Burkina Faso; 4 Université Ouaga II, Ouagadougou, Burkina Faso; 5 Institut Supérieur des Sciences de la Santé (INSSA), Bobo-Dioulasso, Burkina Faso

**Keywords:** Covid-19, riposte, Burkina Faso

## Abstract

Le monde entier est touché par un bouleversement sans précédent, crée par un virus incontrôlable et qui a pris le pas sur les théories scientifiques les plus élaborées. Les grandes puissances peinent à empêcher l’hécatombe dans les effectifs de leurs citoyens infectés, en dépit de toutes les avancées scientifiques et technologiques. Les pays à ressources limitées et dans lesquels vivent des populations parmi les plus vulnérables apparaissent comme les cibles sur lesquelles le virus est susceptible de faire le maximum de dégâts. Cette note discute des approches stratégiques, propose des mesures politiques et suggère des recommandations. La capacité de dépistage/diagnostic, les mesures de protection et d’assainissement, la communication et l’implication de la communauté seraient des priorités de riposte.

## Opinion

La première question à se poser à l’annonce d’une décision de confinement était de connaître auparavant le nombre de personnes contaminées si le résultat du test au coronavirus (COVID-19) revenait positif. Avec l’espoir d’être indemne de cet épisode, la deuxième question était d’estimer le risque encouru à la reprise des activités professionnelles ou de première nécessité. Le but de la présente note est de contribuer à éveiller la conscience de citoyen, de chaque burkinabè quel que soit son niveau d’instruction, son appartenance politique, religieuse ou ethnique, et son niveau de hiérarchique en famille, en communauté ou en milieu professionnel. Convaincu que la pierre angulaire de cette guerre asymétrique reste l’éveil de conscience de toutes et de tous, il est à relever que «Nul n’est à l’abri». La prise de conscience ne pourrait aucunement être attribuée uniquement aux politiques et décideurs. Tout burkinabè devrait prendre conscience du danger encouru et alors l’unique et essentielle question qui s’impose à tous serait: que faire? Peut-être, y aura-t-il une plus-value ou non à faire cet exercice, mais nous nous devons au moins d’être guidés par les propos de Franklin Delano Roosevelt qui disait ceci: «Il est dur d’échouer mais il est pire de n’avoir jamais tenté de réussir».

La présente démarche a été écrite sous le paradigme de la recherche-action qui selon Bazin n’est pas seulement de la recherche et ni seulement de l’action mais la réunion des deux, créant un nouvel espace de travail qui éclaire différemment les situations humaines et donc des problématiques [1, 2]. Cette approche est soutenue par un contexte mondial et scientifique qui impose à la recherche et à l’action d’être concomitants, et où les leçons apprises par les uns devraient concourir à leur bien-être et à celui des autres. La présente note interpelle sur la question du dépistage, de la rupture de la chaine de transmission et de la gestion multisectorielle et intersectorielle de la crise liée au coronavirus au Burkina Faso. Elle discute des perceptions du virus et de la maladie, des approches stratégiques, propose des mesures politiques, et formule des recommandations.

### Perceptions face au Coronavirus

**Quels signes d’alerte considérer:** les primaires ou les secondaires? Un plan de préparation et de riposte à une épidémie de maladie à coronavirus a été élaboré en février 2020 révisé successivement en mars puis en avril 2020. Des directives au niveau national ont été élaborées avec la mise en place d’un comité de coordination. Selon lesdites directives, un cas suspect est défini *«comme un patient qui présente une infection respiratoire aigüe avec toux, difficulté respiratoire et fièvre (température supérieure à 38°C) et notions de voyage dans un pays au cours des 14 jours précédant l’apparition des symptômes ou étant en contact avec un cas confirmé au COVID-19 ou personne ayant travaillé ou séjourné dans un hôpital/site d’isolement dans lequel un cas d’infection au Covid-19 a été confirmé».* Cependant, les premiers signes de la maladie seraient d’abord les céphalées, les douleurs musculaires et la fatigue; la fièvre et les signes respiratoires survenant secondairement [3]. Cette assertion amène à se poser la question suivante: «Toux, fièvre et gêne respiratoire» ne sont-ils pas des signes d’atteinte pulmonaire à un stade déjà avancé?

La connaissance de l’épidémiologie et de la symptomatologie d’une maladie est incontournable pour la définition de stratégies adéquates de riposte. Selon une étude réalisée à Wuhan [4], la fièvre serait un signe dominant mais non le premier signe de début de la maladie, alors qu’actuellement les signes recherchés sont la fièvre, la toux et la gêne respiratoire. Pour prendre le pas sur le virus, deux suggestions pourraient se faire: (i) recueillir et faire le suivi de tout signe chez les cas contacts et ne pas se focaliser uniquement sur les signes qui ne surviennent que secondairement (fièvre, toux, gêne respiratoire) dans le cas du screening; (ii) faire la recherche des cas contacts secondaires avant que le cas contact primaire ne soit testé positif. Cette dernière suggestion parait être difficile ou utopique mais serait plus efficiente que la gestion d’un plus grand nombre de cas dans un pays à ressources limitées.

**Quels postulats pour rompre la chaîne de transmission?** Selon les directives, il faut un dispositif au niveau des points d’entrée terrestres et aéroportuaires du pays et qui est focalisé sur la fièvre et les signes respiratoires. Des dispositifs donc pour prendre la température ont été mis en place par le ministère de la santé avant la déclaration du premier cas. Cependant, n’aurait-il pas été opportun de mettre également l’accent sur la quarantaine des voyageurs en début de l’ épidémie car l’infection peut être asymptomatique ou pauci symptomatique (entrainer pas ou peu de manifestations cliniques) chez 30 à 60% des sujets infectés [3]. Du reste, l’expérience taiwanaise et de plusieurs autres pays ont montré la pertinence de cette démarche dès le début de l’épidémie de COVID-19.

Aussi partant du postulat que le virus peut vivre sur une durée de trois heures à 5 jours en aérosol et sur les surfaces [5] et cela en dehors de la transmission interhumaine, un autre élément important à considérer est la transmission par contact des surfaces. Le lavage des mains devrait être accompagné du nettoyage fréquent des surfaces. Puisque les nettoyages ne sont souvent pas de rigueur, la quarantaine de 14 jours d’un cas contact pourrait être allongée de cinq jours, considérant que le virus pourrait survivre cinq (5) jours sur les surfaces (dont carton papier billets). Enfin, l’infection peut être asymptomatique ou pauci symptomatique alors que les signes recherchés actuellement sont uniquement ceux de la phase d’état; ne serait-il pas adéquat de se focaliser davantage sur la détection et le dépistage des cas contacts? Le dépistage devient alors essentiel pour rompre la chaine de transmission.

### Quelles approches stratégiques?

**Quelle place de la recherche dans ce contexte?** La recherche scientifique fait partie de la riposte et se met en place dès la phase de préparation. Les insuffisances des mécanismes de partage de données, mises en lumière lors l’épidémie de maladie à virus Ebola (MVE) de 2013-2016 en Afrique de l’Ouest, ont porté la question de l’accès aux données au premier plan des enjeux de santé mondiale [6]. La recherche est une opportunité pour la capitalisation des leçons apprises dans les premiers pays touchés par l’épidémie. Aussi, elle peut faciliter la contextualisation de certaines directives adoptées dans un cadre international. Les efforts visant à accélérer la communication des données et des résultats ne doivent pas se limiter aux essais cliniques, mais inclure les études d’observation, la recherche opérationnelle, la surveillance de routine et les informations relatives au virus et à ses séquences génétiques, ainsi que le suivi des interventions de lutte contre la maladie [7]. Il n’est pas de trop d’insister sur le fait que la riposte est la réponse à l’épidémie en cours.

**Quelle approche de management et quel leadership pour la gestion de la crise?** L’approche multisectorielle, multidisciplinaire et participative se doit être la voie incontournable pour la gestion de cette crise. Les autres secteurs ministériels devraient assurer une fonction support en s’associant fortement à la préparation, la gestion; en apportant leurs technicités et la mise à disposition d’équipement, de matériel et de consommables sous la coordination d’un niveau de pilotage le plus élevé possible comme le cas au Burkina Faso avec le premier ministère. Les Organisations de la société civile (OSC) seraient une fois de plus un pivot central de la lutte contre cette nouvelle épidémie dans le contexte du Burkina Faso où subsistent des pesanteurs socioculturelles sur les questions de santé. L’Information-Education-Communication/Communication pour le Changement de Comportement (IEC/CCC) est également un gros maillon pour l’attente d’un sens commun où tous les acteurs auront une même compréhension et un même langage. Il serait opportun d’activer la gouvernance locale avec au centre des préoccupations, la communauté encadrée, orientée et informée par les agents de santé et les relais communautaires. Cela permettra l’empowerment pour un comportement individuel et collectif responsable et favorable à la limitation de la contamination.

**Quelle est la première étape pour rompre la chaine de contamination?** Il faut renforcer les capacités de dépistage. La chaine de transmission serait probablement bientôt non maitrisable avec la perte du lien épidémiologique et partant des postulats que la fièvre n’est qu’un signe de la phase d’état et de la théorie de la transmission par les surfaces; il est plus que nécessaire de renforcer les capacités de dépistage. Il faut noter que selon le rapport de la situation N° 23 identifiant sur 451 appels, seulement 24 alertes qui ont été considérées. Le rapport N° 20 indique que sur 108 appels seulement 10 alertes ont été considérées [8]. Cette faible détection des alertes suscite des questions sur le processus et l’éligibilité de la déclaration d’alerte et sur le risque de dissémination de la maladie dans la population suite à une sous notification. Un dépistage rapide et étendu serait donc nécessaire pour la riposte au Covid-19. Il aurait pour résultat un nombre réel de cas dépistés, ses effets seraient un nombre réel de personnes malades et cas contacts pris en charge et son impact serait la rupture de la chaîne de transmission.

**Figure 1:**
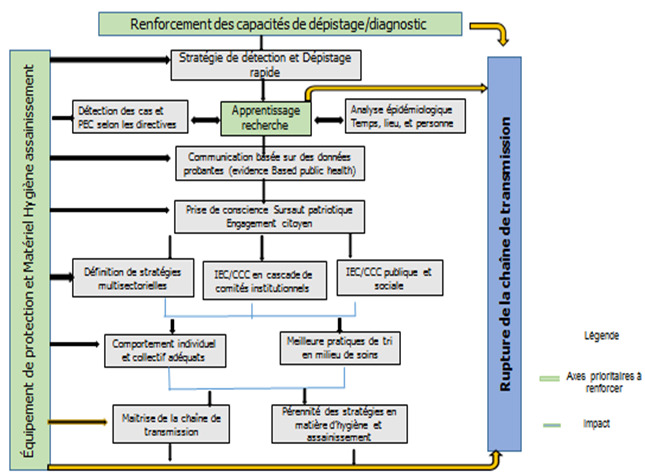
Modèle de gestion multisectorielle de la crise au Covid-19 au Burkina Faso

**Quelles mesures politiques?** Une crainte est que cette pandémie ne devienne une des diverses maladies infectieuses à profil africain caractérisé par un niveau d’hygiène et d’assainissement précaire. Les pays africains, notamment ceux de l’Afrique de l’Ouest, au-delà de la riposte actuelle, sont davantage interpellés à concentrer davantage leurs efforts sur l’hygiène et l’assainissement. Il existe quatre scenarios pour orienter la stratégie de riposte selon l’OMS [9]: (i) zéro cas, (ii) cas sporadiques, (iii) grappes de cas, (iv) transmission communautaire. Il reste qu’il faut opter pour un scenario donné alors qu’il y a une faible capacité de dépistage. Comment faire une analyse descriptive selon le temps, le lieu et les personnes sans dépistage? Dans le contexte du Burkina Faso, la promotion de mesures d’hygiène est réalisée à travers les médias; des mesures de distanciation sociale sans aller au confinement total sont également adoptées. Au-delà de ces mesures, trois axes stratégiques devraient être consolidés: le dépistage, l’équipement-matériel-consommable, et l’apprentissage. Ces trois axes prioritaires sont présentés dans la Figure 1 illustrant un modèle conceptuel de coordination et collaboration multisectorielle et intersectorielle.

Une bonne stratégie de dépistage rapide et étendu au maximum de cas contact pourrait être le point de départ d’une meilleure gestion des cas et de l’exposition du personnel de santé d’une part; d’autre part cette stratégie constituerait une base pour la recherche, la capitalisation des leçons apprises et les analyses épidémiologiques. Les productions faites à partir de la recherche devraient orienter et permettre la production d’informations scientifiques en adéquation avec le contexte local. La stratégie de communication devrait toucher toutes les couches sociales du pays et requérir le concours de tous: acteurs de tous les secteurs ministériels, du privé, des décideurs, des autorités coutumières et religieuses, des organisations de la société civile. Elle devrait susciter un changement de comportements individuels et collectifs en faveur de bonnes pratiques d’hygiène d’une part et d’autre part une action coordonnée et solidaire de tous les secteurs. A cet effet la prise en charge des patients, la gestion de l’information sanitaire et le dépistage pourraient être déconcentrées. Des stratégies au niveau communautaire impliquant les agents de santé communautaire seraient indispensables en ce qui concerne la communication, la gestion des rumeurs et les pratiques des communautés en cas de décès. Plusieurs interventions pourraient être produites à partir de la présente logique conceptuelle. Une attention particulière pourrait être portée à la gestion des décès dans un contexte de transmission communautaire, à la réflexion sur la place et la formation des techniciens de surface (garçon de salle, fille de salle) et à la gestion de l’information sur les décès en communauté.

Le renforcement en équipement de protection, en matériels et consommables en hygiène et assainissement ne se discute plus de nos jours. Des initiatives de productions locales devraient être saluées et encadrées par d’autres secteurs ministériels en dehors de la santé. Il est à souligner que toutes ces mesures politiques proposées nécessitent et ne pourraient être mises en œuvre sans un renforcement de la coordination et la collaboration intersectorielle d’où un ajustement du plan de préparation et de riposte dans lequel les acteurs doivent aller au-delà de ceux d’un comité de gestion d’une crise sanitaire, ou d’un ministère de la santé. Cette gouvernance alliée à une bonne stratégie de communication au sein des communautés faciliterait une maîtrise de la chaine de transmission et une pérennité de bonnes pratiques en hygiène et assainissement aboutissant alors à une rupture de la chaîne de transmission. A partir de ces différentes assertions émises, des recommandations sont suggérées selon une approche multisectorielle et présentées dans le Tableau 1. Au titre du renforcement des capacités de dépistage/diagnostic et de prise en charge, l’attention pourrait être attirée sur les recommandations suivantes: (i) mettre à profit les laboratoires disposant du Gen Xpert pour le dépistage dans les régions sanitaires [10] (ii) adapter la définition de cas contact selon le niveau de transmission de l’OMS (ii) disposer de test de dépistage (iv) mettre en place une gestion de l’information sur les décès en communauté, (v) déconcentrer la prise en charge des cas (vi) renforcer la gestion sécurisée des décès en communauté et en milieu de soins. Promouvoir la fabrication de gel hydro-alcoolique, alcool, de savon liquide et/ou solide et eau de javel, de matériel d’hygiène et assainissement, des équipements de protection et renforcer l’accessibilité de l’eau aux populations sont des actions capitales pour un renforcement en équipement protection et Matériel Hygiène assainissement. Pour assurer un changement pérenne de comportement en matière d’hygiène et assainissement, il serait essentiel de: (i) renforcer les capacités des instituts de recherche en santé publique dans la production de données probantes, (ii) renforcer l’IEC/CCC en hygiène assainissement dans les services de santé cliniques et para cliniques, les lieux de travail et en communauté, (iii) promouvoir les bonnes pratiques en hygiène et assainissement dans les services de santé, aux lieux de travail et en communauté.

**Tableau 1:** Recommandations suggérées pour renforcer la riposte à l’épidémie de Covid-19

Recommandations	Ministères/Institutions concernées
Renforcement des capacités de dépistage/diagnostic et de prise en charge	
Mettre à profit les laboratoires ayant un Gen Xpert pour le dépistage dans chaque région sanitaire	Gouvernement, Ministère de la santé, Ministère de la recherche, Partenaires au développement
Adapter la définition de cas contact selon le niveau de transmission de l’OMS	Conseil scientifique, Ministère de la santé, Partenaires au développement
Disposer de test de dépistage	Gouvernement, Ministère de la santé, Ministère de l’Economie, Partenaires au développement
Former les agents des laboratoires des régions sur le diagnostic	Ministère de la santé
Mettre en place une gestion de l’information sur les décès en communauté	Ministère de la santé (tous les niveaux de la pyramide), Collectivités, Ministère chargé de l’action sociale
Etablir une cartographie des décès	Ministère de la santé, Collectivités
Déconcentrer la prise en charge des cas	Ministère de la santé, Ministère chargé de l’action sociale
Développer la gestion active des cas	Ministère de la santé (tous les niveaux de la pyramide sanitaire) OSC
Renforcer la gestion sécurisée des décès en communauté et en milieu de soins	Ministère de la santé (tous les niveaux de la pyramide sanitaire), Collectivités, Communautés
Renforcement en équipement protection et Matériel Hygiène assainissement	
Promouvoir la fabrication de gel hydro-alcoolique, alcool et eau de javel	Ministère de la recherche Ministère de la santé
Promouvoir la fabrication de matériel d’hygiène et assainissement	Ministère de l’industrie Ministère du commerce
Promouvoir la fabrication des équipements de protection	Gouvernement, Ministère du commerce
Promouvoir la fabrication de solution savonneuse, de savon	Ministère de la jeunesse, Ministère de l’administration territoriale, OSC
Renforcer l’accessibilité de l’eau aux populations	Ministère de l’eau et assainissement, Ministère de la santé
Renforcer l’accessibilité à un assainissement sain aux populations	Ministère de l’eau et assainissement, Ministère de l’administration territoriale
Communication basée sur des données probantes pour un changement pérenne de comportement en matière d’hygiène et assainissement	
Renforcer les capacités des instituts de recherche en santé publique	Ministère de la santé (INSP), Ministère de la recherche, Partenaires au développement
Renforcer l’IEC/CCC publique en hygiène assainissement	Ministère de la communication (média et sociétés de téléphonie), Ministère de la culture la santé
Renforcer IEC/CCC auprès des communautés	Leaders religieux et coutumiers, Comité institutionnel, administratif, Ministère de la santé
Renforcer IEC/CCC auprès des populations	OSC, Agents communautaires, Personnel de santé, Collectivités, Autorités administratives
Rendre disponible à temps réel auprès des agents de santé les directives de précaution et de tri des patients	Ministère de la Santé, Comité de coordination de la crise au Coronavirus Comité scientifique
Promouvoir les bonnes pratiques en hygiène et assainissement	Ministère de la santé, Ministère de l’eau, Collectivités territoriales, OSC
Promouvoir les bonnes pratiques en hygiène et assainissement dans les services de santé	Responsables de structures publiques, privées de santé dont les officines et tradi-praticiens
Promouvoir les bonnes pratiques en hygiène et assainissement	Responsable du secteur formel et informel, Tous
Briefer les agents de santé du public, privé et tradi-praticiens sur directives de prise en charge du covid-19	Ministère de la santé
Appliquer les mesures de prévention sur le coronavirus	Tous les ministères, Tout agent de santé, Population
Réorganiser les services pour la prise en charge des cas	Ministère de la santé, Chef de service (public, privé, clinique, paraclinique)

La lutte contre le Covid-19 dans les pays en développement, en particulier au Burkina Faso, devrait avoir deux buts ultimes: une maitrise de la chaîne de transmission et une pérennité des stratégies en matière d’hygiène et assainissement. Ces deux concepts devraient être des alliés dans notre contexte, car au-delà d’une éventuelle découverte thérapeutique et de dépistages validés scientifiquement, les pays à ressources limitées ayant un système de santé fragile doivent investir prioritairement dans la prévention.

## Conflits d’intérêts

Les auteurs ne déclarent aucun conflit d´intérêts.
